# Machine learning-based nomogram predicts heart failure risk in elderly relapsed/refractory multiple myeloma patients receiving carfilzomib-based therapy

**DOI:** 10.3389/fonc.2025.1624680

**Published:** 2025-09-03

**Authors:** Dan Qiao, Hai-bin Ding, Cong-hui Zhu, Ren-an Chen, Lei Nie

**Affiliations:** ^1^ Department of Medical Oncology, Shaanxi Provincial Cancer Hospital, Xi’an, Shaanxi, China; ^2^ Department of Hematology, Xi’an Daxing Hospital, Xi’an, Shaanxi, China

**Keywords:** multiple myeloma, carfilzomib, heart failure, nomogram model, machine learning

## Abstract

**Objective:**

To develop and validate a machine learning-based nomogram for predicting heart failure (HF) in elderly patients with relapsed/refractory multiple myeloma (RRMM) receiving carfilzomib-based therapy, facilitating early identification and individualized clinical management.

**Methods:**

This retrospective study analyzed clinical data from 192 elderly RRMM patients treated with carfilzomib-based therapy at Shaanxi Provincial Cancer Hospital (from January 1, 2023, to December 31, 2024). Machine learning algorithms, including the Least Absolute Shrinkage and Selection Operator (LASSO) regression, Support Vector Machine (SVM), and Extreme Gradient Boosting (XGBoost), were used for variable selection. Robust predictors identified through cross-model consistency evaluation and bootstrap resampling were incorporated into a nomogram. Model performance was assessed using concordance index (C-index), calibration curves, and decision curve analysis (DCA).

**Results:**

HF occurred in 25.5% (49/192) of patients. Machine learning models consistently identified coronary artery disease (CAD), hypertension, renal insufficiency, and albumin (Alb) levels as significant HF risk factors. The nomogram showed good predictive performance (C-index: 0.780, 95% CI: 0.704–0.841), internal calibration (Hosmer–Lemeshow χ² = 1.334, *P* = 0.970), and external validation (Hosmer-Lemeshow χ² = 1.054, *P* = 0.788). DCA confirmed clinical utility across a wide range of threshold probabilities (1% to 83%), with a peak net benefit of 0.248.

**Conclusion:**

This study provides a practical nomogram for cardiovascular risk assessment in elderly RRMM patients receiving carfilzomib-based therapy, which may assist clinicians in early risk stratification and support tailored monitoring and management throughout treatment.

## 1 Introduction

Multiple myeloma (MM) is a hematologic malignancy characterized by the proliferation of clonal plasma cells in the bone marrow and associated organ dysfunction (1). As the second most common hematologic malignancy, MM accounts for approximately 1.0% of all cancers and 13.0% of hematologic malignancies, with a median age at onset of 65 years and a 1.2-fold higher incidence in males than in females (2). Age-related frailty status and underlying comorbidities, especially cardiovascular and renal diseases, critically affect tolerability to multiple drug combinations or transplantations in elderly patients. Although novel agents, particularly proteasome inhibitors (PIs) and immunomodulatory drugs (IMiDs), have markedly improved outcomes, MM remains incurable. Most patients eventually progress to relapsed/refractory multiple myeloma (RRMM) over the course of their disease. Upon relapse, patient prognosis progressively deteriorates with successive lines of therapy, driven by escalating drug resistance and cumulative disease- and therapy-related complications (3).

Carfilzomib is a second-generation selective PI that irreversibly binds to the active sites of the 20S proteasome and the core component of the 26S proteasome (4), leading to substrate accumulation, tumor cell apoptosis or growth arrest (5). Carfilzomib received accelerated approval from the U.S. Food and Drug Administration (FDA) in 2012. In 2021, the National Medical Products Administration (NMPA) of China approved the combination of carfilzomib, lenalidomide, and dexamethasone (KRd) regimen for the treatment of RRMM patients who have received at least two prior lines of therapy. In clinical practice, KRd and carfilzomib plus dexamethasone (Kd) are preferred in China due to their lower cost, wider insurance coverage, and more convenient twice-weekly dosing schedule. In contrast, carfilzomib, pomalidomide, and dexamethasone (KPd), as well as daratumumab, carfilzomib, and dexamethasone (D-Kd) are less accessible due to limited reimbursement coverage, greater out-of-pocket expenses, and strict weekly administration schedules that may be difficult for elderly patients to adhere to over time. Although the NCCN Clinical Practice Guidelines in Oncology: Multiple Myeloma (2024 Version 2) have elevated carfilzomib to first-line therapy for newly diagnosed multiple myeloma (NDMM), its primary clinical application in China remains focused on RRMM.

Carfilzomib-based combinations have significantly improved outcomes in RRMM, with a 9-month extension in median progression-free survival (PFS) and 8-month overall survival (OS) advantage over standard therapies like lenalidomide plus dexamethasone (Rd) and bortezomib plus dexamethasone (Vd) (6, 7). Despite its potent therapeutic efficacy in MM treatment, carfilzomib is consistently associated with a significantly increased risk of cardiovascular adverse events (CVAEs), including heart failure (HF), hypertension, arrhythmia, ischemic events, dyspnea, and peripheral edema, compared to non-carfilzomib regimens (6, 8). Approximately 51.0% to 57.6% of MM patients receiving carfilzomib experienced one or more CVAEs (9, 10), with a median time to onset of 3.1 months after treatment initiation (10). A meta-analysis demonstrated a 2.3-fold increased risk of all-grade HF with carfilzomib administration in MM patients, which persisted even after adjustment for treatment duration (11). Clinical trials reported 8.2%–10.7% incidence of HF associated with carfilzomib (12, 13), but its actual incidence in real-world settings is likely higher. A cohort study of 815 carfilzomib-treated MM patients identified a 16.2% HF occurrence with a median onset at 4.3 months (10). Another investigation found that 22.7% (5/22) of MM patients experienced left ventricular failure, and grade 3/4 adverse events accounted for 50.0% (14). Importantly, existing studies often do not distinguish between *de novo* acute heart failure (AHF) and acute decompensated heart failure (ADHF), leaving a gap in understanding the specific triggers and risk profiles for each scenario in the context of carfilzomib exposure.

However, most evidence on carfilzomib-related cardiovascular risks originates from Western populations, with limited data specific to Chinese cohorts due to its later regulatory approval in China. A Chinese single-arm trial of twice-weekly Kd in 123 heavily pretreated RRMM patients (median age 60 years) reported grade ≥3 adverse events in 76.4% of participants, including hypertension (13.8%) and cardiac failure (0.8%) (15). Conversely, a Japanese multicenter retrospective study of 157 patients receiving carfilzomib-based regimens (KRd: n = 107; Kd: n = 50) demonstrated differential cardiovascular risks: the KRd cohort showed a 9.3% incidence of grade ≥3 HF, representing a 2.3-fold increase compared to the 4% rate observed in the Kd group (16). These variations may reflect differences in baseline characteristics and clinical practices across study populations.

Given the elevated HF burden in RRMM patients receiving carfilzomib-based therapy, current risk stratification is limited due to the absence of predictive models that account for both cardiovascular comorbidities and myeloma treatment regimens. This study specifically focuses on a cohort of elderly RRMM patients initiating carfilzomib with preserved baseline cardiac function (left ventricular ejection fraction, LVEF ≥55%), a clinically relevant population where unexpected cardiotoxicity poses a significant management challenge. While patients with stable, mild chronic heart failure (CHF), classified as New York Heart Association (NYHA) class I/II, were included to reflect real-world practice, this study aims to develop a nomogram that integrates routine baseline characteristics and treatment-related variables to estimate pre-treatment HF risk, primarily applicable to patients with preserved systolic function. By converting diverse clinical data into simple risk categories, this tool aims to improve understanding of cardiovascular risks and inform treatment decisions, supporting the development of tailored cardioprotective strategies for elderly myeloma patients initiating carfilzomib-based therapy.

## 2 Methods

### 2.1 Study population

We retrospectively analyzed clinical data from 192 RRMM patients treated with carfilzomib-based therapy at Shaanxi Provincial Cancer Hospital between January 1, 2023, and December 31, 2024. Inclusion criteria included: (1) Age ≥60 years with a confirmed diagnosis of RRMM according to the NCCN Clinical Practice Guidelines in Oncology: Multiple Myeloma (2023 Version 1); (2) Treatment with carfilzomib-based therapy, including KRd, Kd, KPd, and D-Kd; (3) Diagnosis of HF based on the 2022 AHA/ACC/HFSA Guideline for the Management of Heart Failure (17), including *de novo* AHF or ADHF, confirmed by clinical evaluation, echocardiography, and pro-B-type natriuretic peptide (NT-proBNP) levels (≥900 pg/mL for patients 50–75 years, ≥1800 pg/mL for patients >75 years), with independent validation by two cardiologists; (4) Preserved cardiac function, defined as LVEF ≥55%, measured by transthoracic echocardiography at baseline; (5) Prior treatment with at least two lines of therapy, including PIs and IMiDs. Exclusion criteria included: (1) POEMS syndrome; (2) Plasma cell leukemia; (3) Prior treatment with carfilzomib; (4) Concurrent malignancies; (5) Pre-existing AHF or CHF with NYHA class III or IV; (6) Inadequately managed cardiovascular conditions, including uncontrolled hypertension, refractory arrhythmias, or severe valvular heart disease.

This study was approved by the Medical Ethics Committee of Shaanxi Provincial Cancer Hospital. All participants provided written informed consent for the use of their clinical data exclusively for research purposes.

### 2.2 Patient baseline characteristics

Baseline characteristics included age, sex, ECOG PS (Eastern Cooperative Oncology Group performance status), and body mass index (BMI). Myeloma-related factors were assessed using the Durie-Salmon Staging System (DSS), International Staging System (ISS), prior anthracycline use, stem cell transplantation, and extramedullary disease (EMD). Laboratory parameters, such as hemoglobin (Hb), albumin (Alb), and lactate dehydrogenase (LDH), were also analyzed. Other clinical comorbidities, including hypertension, coronary artery disease (CAD), CHF, chronic obstructive pulmonary disease (COPD), diabetes, and renal insufficiency (defined as serum creatinine >177.0 μmol/L), were documented.

#### 2.2.1 Variable transformation and categorization

Age was categorized as 60–69, 70–79, and ≥80 years. ECOG PS was grouped as 1, 2, or 3. Hypertension was graded as normal, grade 1, 2, and 3 based on the 2020 International Society of Hypertension Global Hypertension Practice Guidelines (18). For analysis, ECOG scores 1–2 were combined as low-risk (score 0) and score 3 as high-risk (score 1). Hypertension grades 0–1 were defined as low-risk (score 0) and grades 2–3 as high-risk (score 1). LDH, Alb, and Hb were dichotomized using cut-off values determined by receiver operating characteristic (ROC) curve analysis.

### 2.3 Study design

Carfilzomib-based therapy was administered as follows: (1) Kd: Carfilzomib (20 mg/m² on days 1 and 2 of cycle 1; 27 mg/m² thereafter) was administered as a 30-minute intravenous infusion twice weekly (days 1, 2, 8, 9, 15, 16) in 28-day cycles. Dexamethasone 20 mg was given orally on days 1, 2, 8, 9, 15, 16, 21, and 22. (2) KRd: The Kd regimen combined with oral lenalidomide 25 mg (days 1–21). (3) KPd: The Kd regimen combined with oral pomalidomide 4 mg (days 1–21). (4) D-Kd: The Kd regimen combined with daratumumab (8 mg/kg intravenously on days 1–2 of cycle 1 and at 16 mg/kg weekly for the remaining doses of the first two cycles, then every 2 weeks during cycles 3–6, and monthly thereafter) (19). For patients aged >75 years in the D-Kd group, dexamethasone was reduced to 10 mg.

Pre-treatment assessments included a complete blood count, liver and renal function tests, and an electrocardiogram (ECG). Patients presenting with symptoms of cardiac dysfunction (e.g., dyspnea, fatigue, or fluid overload) required further assessment, including echocardiography, chest CT, and biomarkers such as troponin and NT-proBNP. If HF was confirmed, chemotherapy was discontinued immediately, and guideline-directed medical therapy was initiated. Decisions regarding continuation, dose adjustment, or replacement of carfilzomib-based therapy depended on a comprehensive reassessment of the patient’s clinical and cardiac status.

### 2.4 Machine learning models

Three machine learning algorithms were implemented to identify predictive features: least absolute shrinkage and selection operator (LASSO), linear support vector machine (SVM), and extreme gradient boosting (XGBoost).

All candidate variables were transformed into binary categories prior to modeling, based on clinical guidelines or ROC-derived thresholds, as detailed in Section 2.2.1. This approach ensured all variables were standardized for fair comparison and consistent interpretation across algorithms.

LASSO, an L1-penalized logistic regression method, was used for automatic feature selection by shrinking the coefficients of less informative variables to zero. It also reduces model complexity by retaining one representative variable among correlated predictors. The optimal regularization parameter (λ) was determined by 10-fold cross-validation with the one-standard-error rule, which selects the simplest model within one standard error of the minimum prediction error. Before modeling, all variables were screened for multicollinearity using variance inflation factor (VIF) analysis, and all VIFs were below 2, indicating no meaningful collinearity among predictors.

Linear SVM was applied as a margin-based classifier suitable for small samples and low-dimensional settings. Feature importance was inferred from the absolute magnitude of model coefficients, reflecting each variable’s contribution to the classification margin. Nonlinear kernels (e.g., RBF) were excluded to ensure interpretability and stability across methods. Given that all predictors were dichotomized, and collinearity was minimal, linear SVM was considered appropriate for feature selection in this context.

XGBoost, a gradient-boosted decision tree ensemble, was used with L2 regularization (λ = 1) to control model complexity and hyperparameter tuning via grid search. Key hyperparameters included a learning rate (η) of 0.1, maximum tree depth of 5, and subsampling ratios for instances (80%) and features (80%). The algorithm used 5-fold cross-validation with early stopping (15 consecutive non-improvement rounds) to determine optimal iterations (n = 45), balancing computational efficiency and generalizability.

### 2.5 Outcome measures

The primary outcome was the development of HF during carfilzomib-based treatment. Analytical steps included: (1) Comparison of baseline characteristics between patients with and without HF to identify statistically significant differences. (2) Feature selection using three machine learning models, namely LASSO, linear SVM, and XGBoost, to identify potential HF predictors. (3) Variable selection followed a two-stage strategy: (a) Candidate features with high predictive contribution were identified by visualizing Min-Max normalized importance scores in a heatmap (20); (b) Their selection stability was evaluated via bootstrap resampling (100 iterations), with consistently selected variables retained based on boxplot analysis (21). (4) A nomogram was constructed using the final selected variables, and its stability and clinical utility were evaluated via calibration curves and decision curve analysis (DCA). The assignment of point values to each predictor in the nomogram was based on the regression coefficients (*β*) from the final multivariable logistic regression model. The variable with the largest absolute *β* was set to 100 points, and other variables were scaled proportionally. The total score was mapped to predicted HF probability via the logistic function:


$$\text{Risk of HF}=\frac{1}{1+e^{-linear\;predictor}}$$

**Figure 1:**
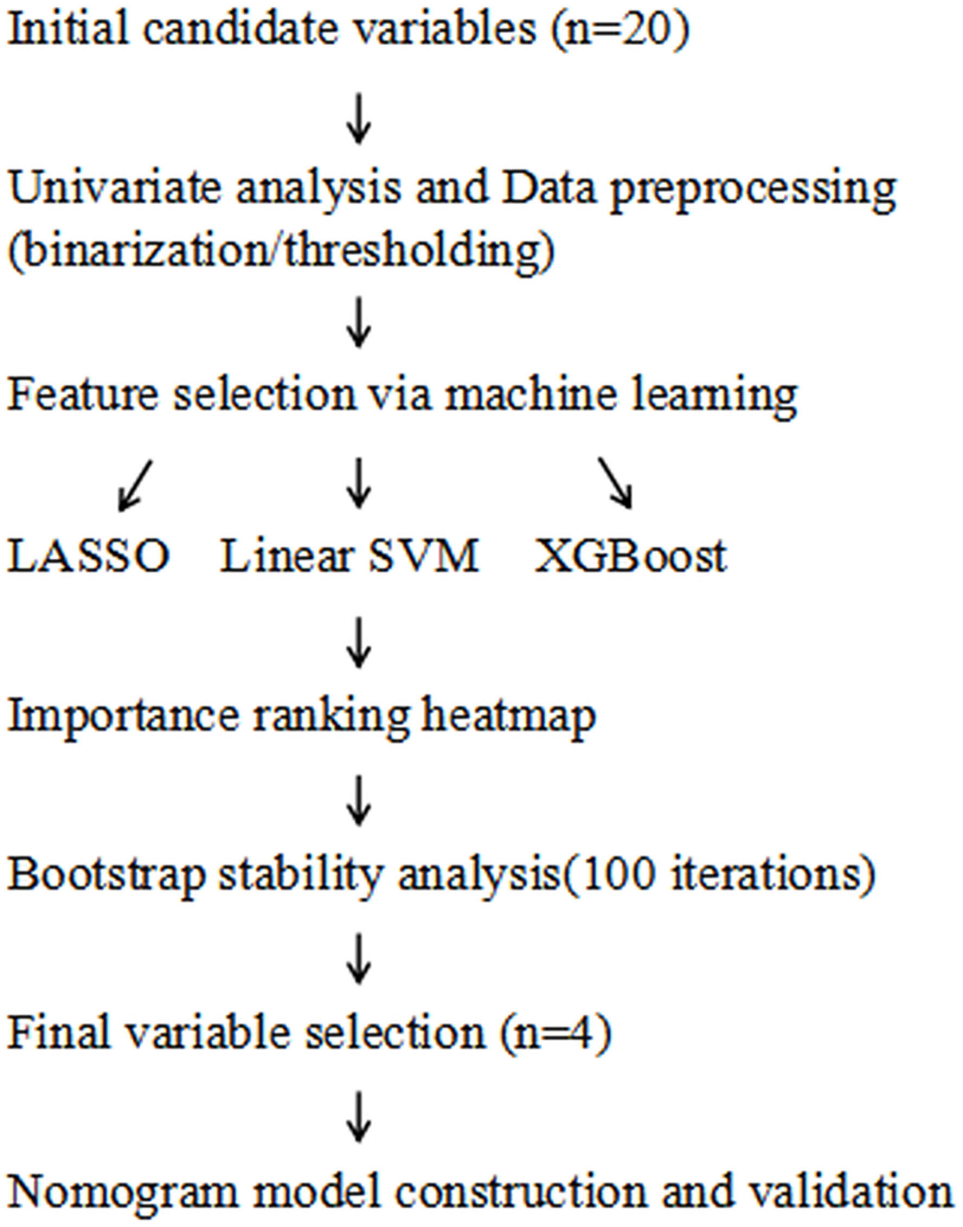
Workflow of variable selection and nomogram model construction. LASSO, least absolute shrinkage and selection operator; SVM, support vector machine; XGBoost, extreme gradient boosting.

(5) External validation was performed using an independent cohort of 65 elderly RRMM patients treated with carfilzomib at our center from January to May 2025. The nomogram’s predictive performance was assessed in this external dataset by calculating calibration curves and the Hosmer-Lemeshow test, as detailed in the Statistical analysis section. DCA was not conducted in the external validation cohort due to the limited sample size, which may lead to unstable net benefit curves, consistent with recommendations in the literature (22). (6) The overall modeling workflow, including variable selection and model validation, was illustrated in **Figure 1**.

### 2.6 Statistical methods

Data preprocessing and initial analysis were performed using SPSS version 27.0 (SPSS Inc., Chicago, IL, USA). Normality was assessed using the Shapiro-Wilk test, and homogeneity of variance using Levene’s test. Normally distributed continuous variables were expressed as mean ± standard deviation and analyzed using the independent samples t-test. Categorical variables were analyzed using the Chi-square test; for ordinal categorical variables, the Mantel-Haenszel chi-square test was applied. Fisher’s exact test was used when any expected cell count was <5. Non-normally distributed continuous variables were analyzed using the Mann–Whitney U test.

Further analyses were conducted using Python (version 3.11.10) and R (version 4.4.3). In Python, core tasks included LASSO coefficient visualization, SVM error rate analysis (scikit-learn 1.2.2), and nomogram clinical utility assessment using DCA (dca 0.0.8). In R, key procedures included LASSO variable selection (glmnet 4.1.8), SVM/XGBoost feature importance ranking (caret 6.0.94, xgboost 1.7.5.1), heatmap and boxplot analysis (ggplot2 3.4.4), stability assessment via bootstrap resampling (boot 1.3.30; 100 iterations), and nomogram construction with calibration curve validation (rms 6.7.0, rms::calibrate). For external validation, the nomogram was applied to the independent cohort to generate predicted probabilities, and calibration was assessed by calibration curves and the Hosmer-Lemeshow goodness-of-fit test (ResourceSelection package).

## 3 Results

### 3.1 Comparison of baseline characteristics

Among 192 elderly RRMM patients treated with carfilzomib-based therapy, 49 (25.5%) developed HF. Clinical characteristics, including performance status, comorbidities, disease stage, and laboratory parameters, were compared between the two groups. Univariate analysis identified ECOG PS (*P* = 0.044), CAD (*P* = 0.006), hypertension (*P* = 0.008), and renal insufficiency (serum creatinine >177.0 µmol/L) (*P* = 0.034) as significant risk factors for HF. In contrast, COPD, CHF with NYHA Class I/II, diabetes mellitus, and EMD were not significantly associated with HF incidence (all *P* > 0.05). Hb, Alb, and LDH levels differed significantly between the two groups (*P* = 0.005, *P* < 0.001, and *P* = 0.019). No significant differences were noted in age, gender, BMI, disease stage (DS and ISS), line of therapy, therapy regimen (KRd, KPd, Kd, or D-Kd), prior anthracycline use, or stem cell transplantation (all *P* > 0.05) (**Table 1**).

**Table 1:** Univariate analysis of HF in elderly RRMM patients receiving carfilzomib-based therapy.

Variables	HF group (n *=* 49)	Non-HF group (n = 143)	*χ* ^2^/*t*	*P*
Age(year)				
60~69	15	64	3.362	0.067
70~79	23	58		
≥80	11	21		
Male	27	83	0.129	0.720
ECOG PS				
1	2	18	4.055	0.044
2	32	96		
3	15	29		
BMI				
Underweight	11	22	0.081	0.776
Normal weight	29	101		
Overweight	9	20		
CAD	33	64	7.451	0.006
COPD	8	27	0.160	0.689
Diabetes	14	43	0.039	0.843
Hypertension				
Normal	10	52	6.950	0.008
Grade 1	11	43		
Grade 2	16	25		
Grade 3	12	23		
Chronic heart failure				
Without	36	116	5.504	0.064
NYHA Class I	4	17		
NYHA Class II	9	10		
DS Stage				
II	7	38	3.055	0.081
III	42	105		
ISS Stage				
I	5	20	2.775	0.096
II	13	55		
III	31	68		
Line of therapy				
3	17	55	1.310	0.252
4	18	62		
≥5	14	26		
Therapy				
KRd	40	86	7.138	0.063
KPd	4	21		
Kd	4	25		
D-Kd	1	11		
EMD	8	34	1.185	0.276
Previous transplantation	6	17	0.004	0.947
Previous anthracyclines use	12	40	0.224	0.636
Renal insufficiency	16	26	4.472	0.034
Hb (g/L)	82.3 ± 10.9	87.6 ± 11.3	2.837	0.005
Alb (g/L)	32.3 ± 3.8	35.2 ± 4.0	4.424	<0.001
LDH (U/L)	318.6 ± 86.4	283.7 ± 90.2	2.358	0.019

HF, heart failure; RRMM, relapsed/refractory multiple myeloma; KRd, carfilzomib, lenalidomide, and dexamethasone; ECOG PS, Eastern Cooperative Oncology Group performance status; BMI, body mass index; CAD, coronary artery disease; COPD, chronic obstructive pulmonary disease; DSS, Durie-Salmon Staging System; ISS, International Staging System; EMD, extramedullary disease; Hb, hemoglobin; Alb, albumin; LDH, lactate dehydrogenase.

### 3.2 Threshold classification of biomarkers

**Figure 2:**
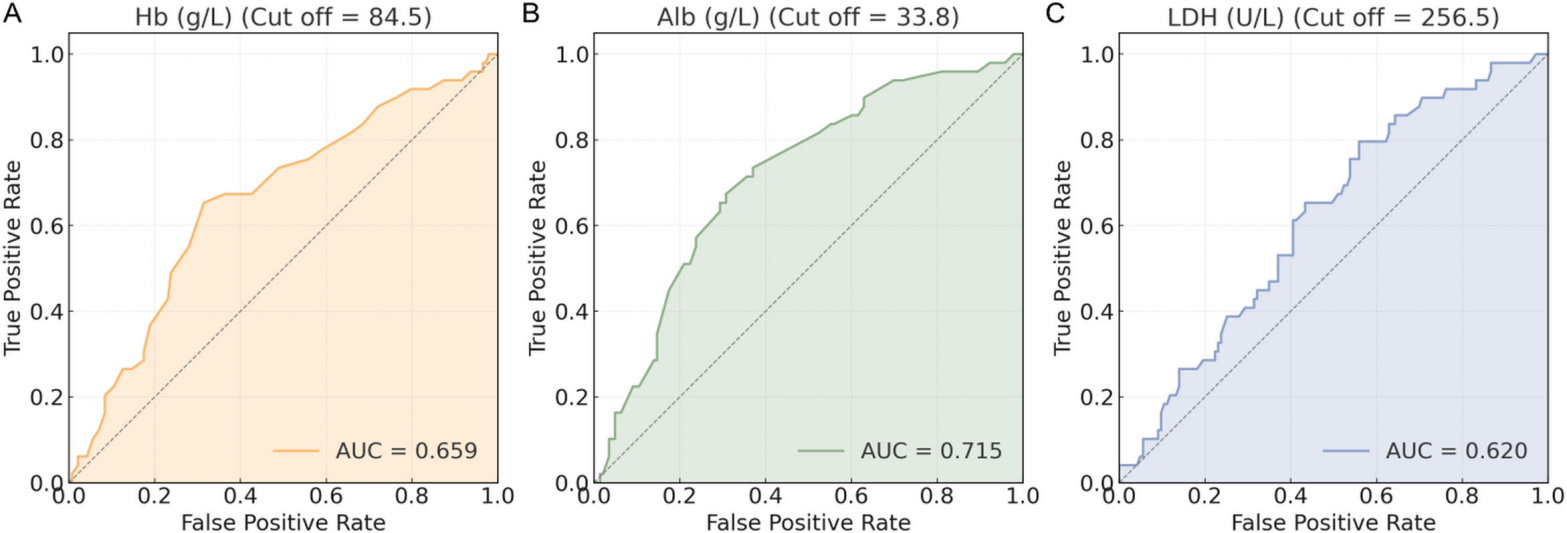
ROC curves of biomarkers. **(A)** Hb (g/L) ROC curve; **(B)** Alb (g/L) ROC curve; **(C)** LDH (U/L) ROC curve. ROC, receiver operating characteristic; Hb, hemoglobin; Alb, albumin; LDH, lactate dehydrogenase.

ROC curves were generated for Hb, Alb, and LDH to evaluate their predictive value for HF (**Figure 2**). Based on Youden index analysis, the optimal cut-off values were 84.5 g/L for Hb, 33.8 g/L for Alb, and 256.5 U/L for LDH. Among these, Alb showed the highest AUC and most effectively discriminated HF risk. The resulting thresholds, along with their corresponding area under the curve (AUC), 95% confidence intervals (CI), sensitivity, specificity, and cutoff values for HF, were systematically recorded (**Table 2**). These cut-off values were subsequently used to dichotomize the biomarkers for further modeling and statistical analysis.

**Table 2:** ROC curve parameters of biomarkers.

Marker	AUC	95% CI	Sensitivity	Specificity	Youden index	Cut off
Hb	0.659	0.570–0.749	0.653	0.685	0.338	84.5
Alb	0.715	0.634–0.796	0.673	0.692	0.366	33.8
LDH	0.620	0.533–0.706	0.796	0.441	0.236	256.5

ROC, receiver operating characteristic; AUC, area under the curve; CI, confidence intervals.

### 3.3 Feature selection via machine learning algorithms

Data showing significant baseline differences were included and assigned according to the outlined criteria (**Table 3**). Three machine learning methods (LASSO, SVM, and XGBoost) were implemented to identify key predictors of HF in elderly RRMM patients undergoing carfilzomib-based therapy.

**Table 3:** Assignment table.

Variables	Assignment content
CAD	Without = 0, With = 1
ECOG PS	1 + 2 = 0, 3 = 1
Hypertension	Normal+Grade1 = 0, Grade2+Grade3 = 1
Renal insufficiency	Without = 0, With = 1
Hb	≥84.5 = 0, <84.5 = 1
Alb	≥33.8 = 0, <33.8 = 1
LDH	≤256.5 = 0, >256.5 = 1

Renal insufficiency: With = serum creatinine >177.0 μmol/L; Without = serum creatinine ≤177.0 μmol/L.

**Figure 3:**
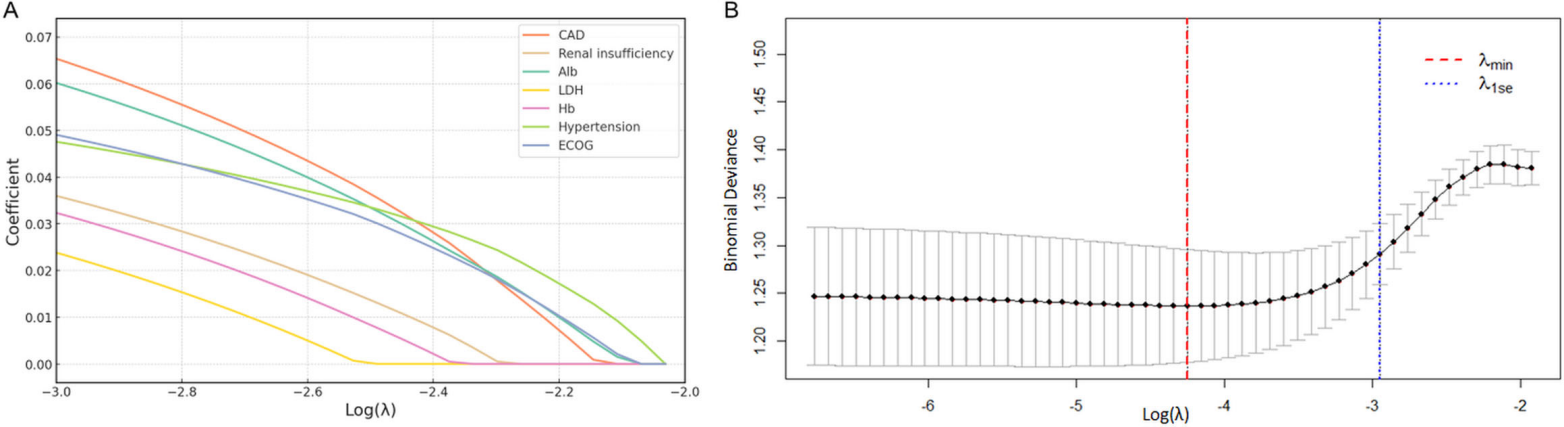
Risk factor screening for HF using LASSO model. **(A)** LASSO regression coefficients for clinical variables; **(B)** Variable selection process via ten-fold cross-validation, with optimal lambda value indicated. LASSO, least absolute shrinkage and selection operator; HF, heart failure.

The LASSO model demonstrated coefficient shrinkage across clinical variables as the regularization parameter (*λ*) increased (**Figure 3A**). CAD (*β* = 0.110) and Alb (*β* = 0.101) demonstrated the strongest associations with HF, while LDH (*β* = 0.062) and Hb (*β* = 0.069) were weaker predictors. The optimal *λ* (*λ*._min_ = 0.014) was identified through cross-validation (**Figure 3B**), balancing model fit and complexity (binomial deviance = 1.237). The one-standard-error rule (*λ*._1se_ = 0.052) provided a simpler model with a slightly higher deviance (1.291), which offered a more conservative and overfitting resistant approach.

**Figure 4:**
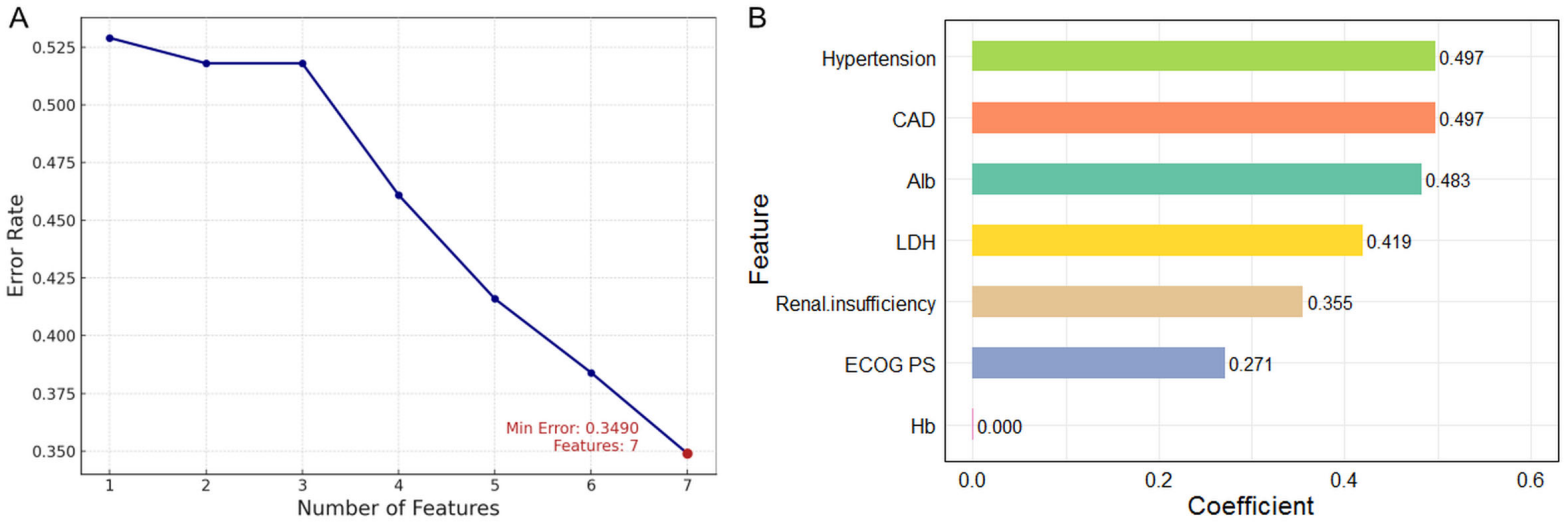
Risk factor screening for HF using SVM model. **(A)** Variation of error rate with number of features; **(B)** Variable importance ranking using linear kernel SVM. SVM, support vector machine; HF, heart failure.

The SVM analysis revealed a progressive reduction in error rates from 52.8% (single feature) to 34.9% (seven features) with feature inclusion with 5-fold cross-validation (**Figure 4A**). Linear kernel analysis indicated hypertension and CAD as primary predictive factors, while Hb showed near-zero contribution (**Figure 4B**).

**Figure 5:**
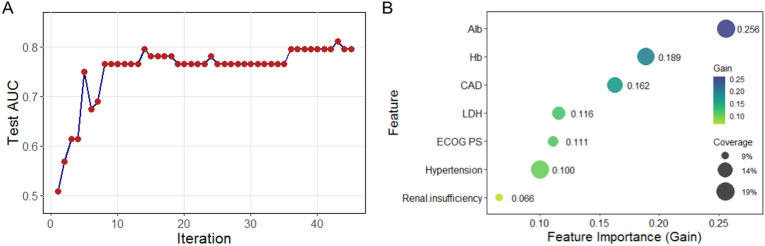
Risk factor screening for HF using XGBoost model. **(A)** Iterative optimization of test AUC during model training; **(B)** Feature importance ranking based on mean decrease in accuracy. XGBoost, extreme gradient boosting; HF, heart failure; AUC, area under the curve.

The XGBoost model showed a progressive increase in Test AUC, reaching a plateau after approximately 10 iterations, with the optimal number of iterations set at 45 (**Figure 5A**). The feature importance plot highlighted that Alb (gain = 0.256, coverage = 19.3%) and Hb (gain = 0.189, coverage = 17.9%) played major roles, along with moderate contributions from CAD, LDH, hypertension, and renal insufficiency (**Figure 5B**).

LASSO, SVM, and XGBoost demonstrated distinct performance profiles (**Table 4**). LASSO achieved the highest AUC (0.805) with balanced sensitivity (0.842) but moderate accuracy (0.730). XGBoost, despite a marginally lower AUC (0.795), excelled in sensitivity (0.909), accuracy (0.824), and F1 score (0.870). SVM exhibited the highest specificity (0.786) but lower sensitivity (0.538) and accuracy (0.667). These disparities highlight the limitations of relying on a single model for variable importance analysis, as each algorithm prioritizes distinct performance aspects.

**Table 4:** Classification performance of LASSO, SVM, and XGBoost models.

Marker	LASSO	SVM	XGBoost
AUC	0.805	0.764	0.795
95% CI	0.718–0.891	0.562–0.936	0.525–1.000
Sensitivity	0.842	0.538	0.909
Specificity	0.647	0.786	0.667
Cut off	0.420	0.392	0.400
Accuracy	0.730	0.667	0.824
Precision	0.640	0.700	0.833
F1 Score	0.727	0.609	0.870

LASSO, least absolute shrinkage and selection operator; SVM, support vector machine; XGBoost, extreme gradient boosting.

### 3.4 Cross-model consistency and stability of feature importance

**Figure 6:**
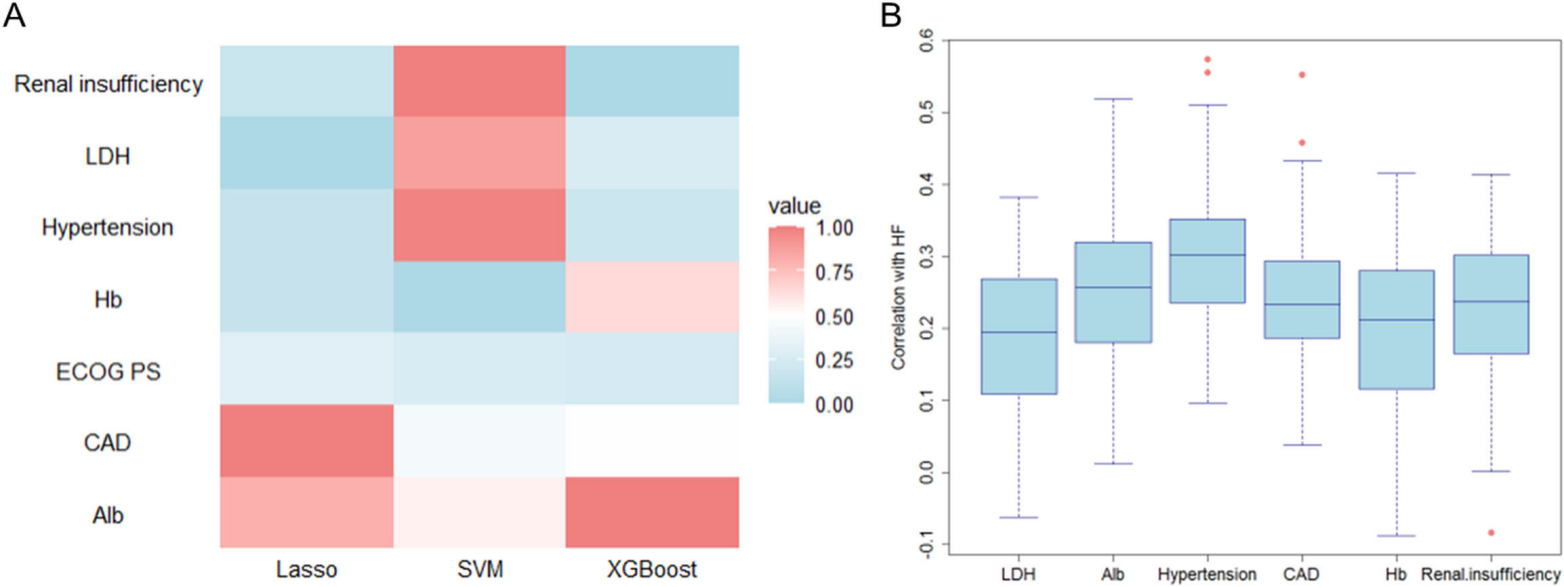
Validation of feature selection consistency across machine learning models. **(A)** Cross-model importance concordance heatmap for LASSO, SVM, and XGBoost; **(B)** Bootstrap-stabilized importance distribution boxplots.

The heatmap of feature importance rankings shows strong agreement across LASSO, SVM, and XGBoost models (**Figure 6A**). Renal insufficiency was the top predictor in all models, especially SVM and XGBoost. LDH showed high importance in XGBoost and moderate relevance in others. Alb maintained stable importance across models, particularly in SVM and XGBoost. Hypertension was significant in LASSO and XGBoost, while CAD ranked highly in LASSO and SVM. Due to its moderate relevance in LASSO, Hb was retained for further analysis, while ECOG PS was excluded because of its consistently low importance in all models.

Variability in importance distributions across the six candidate variables was rigorously evaluated through bootstrap stabilization (**Figure 6B**). Alb demonstrated the most stable importance profile (median = 0.257, IQR = 0.137), followed by hypertension (median = 0.302, IQR = 0.114), CAD (median = 0.234, IQR = 0.106), and renal insufficiency (median = 0.237, IQR = 0.136) (**Table 5**). In contrast, LDH exhibited significantly higher variability (median = 0.195, IQR = 0.159), while Hb showed the widest interquartile range (median = 0.213, IQR = 0.164) and inconsistent contributions across resampling iterations. Based on a predefined threshold of IQR >0.15, both LDH and Hb were excluded due to their limited reproducibility in bootstrap validation.

**Table 5:** Distribution of bootstrap-based feature importance scores.

Variables	Minimum	1st Qu.	Median	Mean	3rd Qu	Maximum
Alb	0.012	0.182	0.257	0.256	0.319	0.519
CAD	0.039	0.187	0.234	0.235	0.293	0.553
Hypertension	0.097	0.237	0.302	0.303	0.351	0.574
Renal insufficiency	-0.083	0.165	0.237	0.229	0.301	0.415
LDH	-0.062	0.109	0.195	0.188	0.268	0.383
Hb	-0.087	0.116	0.213	0.196	0.280	0.415

Qu, Quartile.

### 3.5 Nomogram prediction model construction and validation

**Figure 7:**
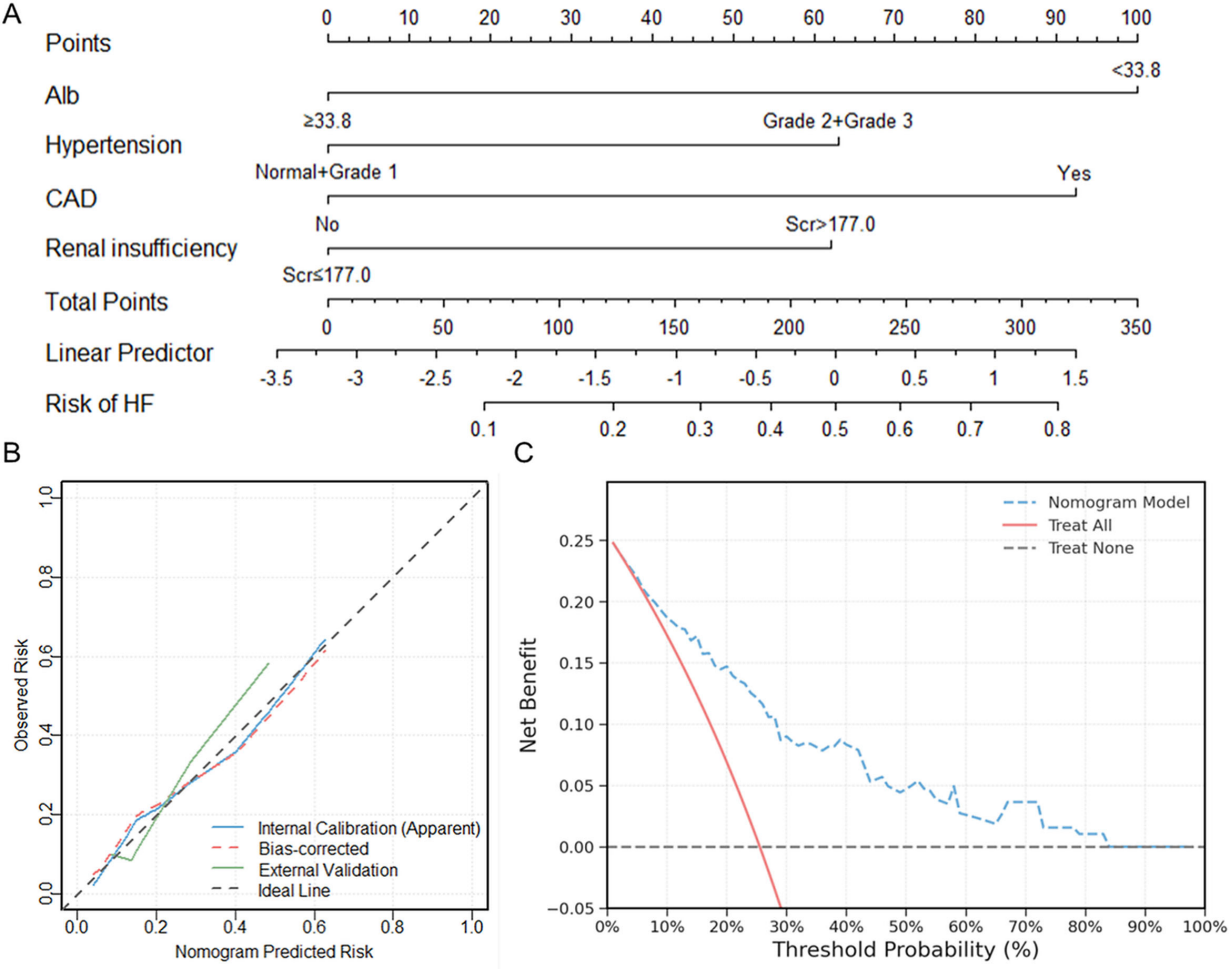
Nomogram model construction and validation for predicting HF in elderly RRMM patients receiving carfilzomib-based therapy. **(A)** Nomogram construction for HF risk stratification; **(B)** Calibration curves comparing predicted and observed HF risk in internal and external cohorts; **(C)** Decision curve analysis showing clinical net benefit across threshold probabilities. HF, heart failure; RRMM, relapsed/refractory multiple myeloma.

The nomogram prediction model for HF in elderly RRMM patients receiving carfilzomib-based therapy was constructed based on Alb, CAD, hypertension, and renal insufficiency (**Figure 7A**). This model achieved a concordance index of 0.780 (95% CI: 0.704–0.841) according to 1000 bootstrap resamples. For clinical application, users sum the points for all variables (as displayed in the nomogram) to yield a total score, which corresponds to the predicted probability of HF. Example risk scores for two representative patients are shown in **Table 6**, demonstrating the practical application of the model.

**Table 6:** Example risk score calculation and predicted probability of HF using the nomogram.

Predictors	HF case	Score	Non-HF case	Score
Alb	27.3	86	35.0	0
CAD	Yes	100	No	0
Hypertension	Grade 2	57	Grade 1	0
Renal insufficiency	Scr ≤177.0	0	Scr >177.0	93
Total score	243		93	
Predicted HF Risk	79.5%		32.9%	

Scr, serum creatinine.

Internal calibration analysis showed good agreement between predicted and observed probabilities, with a non-significant Hosmer-Lemeshow test (χ² = 1.334, *P* = 0.970). External validation using an independent cohort of 65 patients showed good calibration (Hosmer-Lemeshow χ² = 1.054, *P* = 0.788), with the calibration curve intersecting the ideal line across the risk spectrum (**Figure 7B**). DCA demonstrated positive net benefit for the nomogram model across a wide range of threshold probabilities (1% to 83%). The maximum net benefit (0.248) was observed at the 1% threshold, which closely corresponded to the incidence of HF in the cohort (49/192, 25.5%). At the clinically relevant 30% threshold, the model yielded a net benefit of 0.090 (absolute gain of 0.154 compared to the “Treat All” strategy) (**Figure 7C**).

## 4 Discussion

Elderly patients with MM often have significant cardiovascular comorbidities at diagnosis, including previous cardiac events, hyperlipidemia, and diabetes mellitus (23), which complicate therapeutic management in the context of age-related pathophysiological decline. MM patients have a 1.5-fold higher incidence of cardiovascular disease compared to age-matched controls without MM (52.0% vs. 35.0%), especially arrhythmias, HF, cardiomyopathies, and atrioventricular conduction abnormalities (24). Carfilzomib contributes to cardiovascular toxicity through proteasome inhibition in cardiomyocytes, disrupting proteostasis and causing endoplasmic reticulum stress, mitochondrial dysfunction, and cardiomyocyte apoptosis (25, 26). Chemotherapy-induced autonomic neuropathy may potentiate this cardiotoxicity (27). The 2022 ESC Guidelines on cardio-oncology state that pre-existing comorbidities are independent risk factors for carfilzomib-associated CVAEs, highlighting the necessity for comprehensive cardiovascular evaluation before treatment initiation (17).

In our study, HF events included both *de novo* AHF and ADHF. The incidence was 25.5% (49/192), which is higher than the 16.2% (132/815) reported by Bishnoi et al. (10), but similar to the 22.7% (765/3,370) observed in a real-world pharmacovigilance study based on FDA data (28). This disparity likely reflects cohort differences: our study exclusively included RRMM patients, whereas Bishnoi’s cohort contained 43.6% (355/815) NDMM cases (10). In addition, our patients demonstrated a greater prevalence of hypertension (67.7% [130/192] vs. 28.3% [231/815]). The median age of our population was 69 years, with the HF group being older than the non-HF group (72 years vs. 67 years), aligning with cardiovascular epidemiology patterns showing a higher prevalence of HF with increasing age (29). These findings underscore the importance of individualized cardiovascular risk stratification for elderly RRMM patients receiving carfilzomib.

Univariate analysis identified ECOG PS, CAD, hypertension, renal insufficiency, Hb, Alb, and LDH levels as significant risk factors for HF. CHF at baseline (NYHA I/II) was more frequent in the HF group (26.5% vs 18.9%), but did not reach statistical significance in univariate analysis (*P*=0.064) and was therefore not included in subsequent multivariable modeling. This limited predictive value may be related to the relatively low prevalence of CHF, the use of standardized background therapy, and the overall stability of cardiac function at baseline. Current guidelines indicate patients with stable HF who receive guideline-directed medical therapy and have preserved ejection fraction are at lower short-term risk for acute decompensation (30).

Given the above, and to further optimize feature selection, we employed three machine learning methods (LASSO, SVM, and XGBoost), which identified partially overlapping but distinct sets of important predictors. LASSO mainly selected CAD and Alb as the most important predictors, achieving the highest AUC (0.805) and sensitivity (84.2%) but lower specificity (64.7%). Linear SVM ranked hypertension and CAD highly, with the highest specificity (78.6%) but lower sensitivity (53.8%). This may be related to its margin-based linear classification principle and sensitivity to class imbalance (HF:non-HF ≈ 1:2.92). XGBoost prioritized Alb and Hb with the highest sensitivity (90.9%) and accuracy (82.4%). However, its extremely wide 95% CI (0.525–1.000) and moderate specificity (66.7%) suggest overfitting risks in small samples.

These differences in feature selection reflect the distinct statistical assumptions and optimization strategies of each algorithm. LASSO and linear SVM both rely on linear relationships but use different penalties and decision boundaries, while XGBoost can capture nonlinear interactions and higher-order effects, but is more prone to overfitting with limited data. To address these inconsistencies, we combined model-based importance with stability analysis using heatmaps and bootstrap resampling to select final predictors for the nomogram, aiming to balance statistical stability with clinical interpretability. As detailed in the Methods, our variable processing and diagnostic assessment confirmed minimal collinearity and ensured suitability for both LASSO and linear SVM algorithms, thereby supporting the validity of our feature selection and modeling approach.

Heatmap and bootstrap analyses consistently identified renal insufficiency, Alb, hypertension, and CAD as stable predictors. ECOG PS was excluded due to its inconsistent importance across models and its subjective clinical nature. In this elderly cohort, the high rate of ECOG PS ≥2 (89.6%) likely reflected general age-related physiological limitations and anemia-induced functional decline. However, ECOG PS fails to fully represent frailty status, which is defined by meeting 3–5 of the following criteria: weight loss, self-reported tiredness, weakness, slow walking speed, and low physical activity. Previous studies demonstrated that frailty status predicts HF risk more accurately than performance status alone (31). These findings highlight the importance of frailty-based evaluations in assessing HF risk among elderly RRMM patients.

Bootstrap resampling excluded Hb and LDH because of their wide interquartile ranges (IQR >0.15) and instability across iterations. In MM, anemia results from bone marrow infiltration, relative erythropoietin (EPO) deficiency, chemotherapy-induced myelosuppression (32), and nutritional deficiencies (iron, B12, and folate) (33), which increase Hb variability and reduce its reliability as a cardiac risk marker. Elevated LDH levels are associated with poor prognosis in MM and organ dysfunction but lack cardiac specificity (34). Additionally, treatment-related fluctuations further limit its clinical utility for HF prediction (35).

The four retained variables—CAD, hypertension, renal insufficiency, and Alb levels—represent interconnected yet distinct pathophysiological pathways contributing to HF risk in elderly RRMM patients receiving carfilzomib therapy. CAD primarily promotes HF through myocardial ischemia, leading to impaired perfusion, cardiomyocyte necrosis, and cardiac fibrosis, which result in adverse structural and functional cardiac remodeling (36). Carfilzomib exacerbates these effects by activating the NF-κB pathway in cardiac tissue, promoting inflammatory cytokine release (e.g., TNF-α, IL-6) and myocardial fibrosis (26). Moreau et al. reported that twice-weekly carfilzomib administration correlated with increased HF events compared to weekly dosing (37). These cardiotoxic pathways synergize with ischemic injury, accelerating left ventricular systolic dysfunction and diastolic stiffness.

Hypertension induces significant cardiac remodeling, characterized by left ventricular hypertrophy, interstitial fibrosis, and sympathetic nervous system dysregulation, contributing to severe cardiovascular outcomes like ventricular arrhythmias and congestive HF (38). Carfilzomib further increases hypertension risk (10), especially in elderly patients (≥70 years) (39). Notably, carfilzomib could either induce new hypertension or aggravate previously existing hypertension in RRMM patients (40). As a major cardiovascular risk factor of HF in the elderly (41), proactive hypertension management and careful monitoring during carfilzomib therapy remain essential.

MM patients with renal insufficiency have a 2- to 4-fold increased risk of CVAEs (42). Renal insufficiency arises primarily from tubular injury from free light chain deposition, hypercalcemia, and hyperuricemia, which impair glomerular filtration and promote systemic inflammation (43). These processes increase plasma viscosity, exacerbate fluid overload, and electrolyte imbalances (44, 45). Importantly, carfilzomib’s clearance is minimally impacted by renal function, even in dialysis-dependent patients (46, 47), indicating that increased HF risk in renal impairment arises primarily from systemic pathophysiological effects rather than altered drug pharmacokinetics.

Hypoalbuminemia in MM reflects advanced disease stage, malnutrition, renal insufficiency, and chronic inflammation, correlating with poorer outcomes (48). Gopal et al. demonstrated that time-dependent associations between low Alb levels and elevated HF risk in elderly populations (49). Hypoalbuminemia decreases colloid osmotic pressure, promotes interstitial edema, and contributes to diastolic dysfunction, while also activating the renin-angiotensin-aldosterone system and driving ventricular remodeling (50). These mechanisms interact with carfilzomib-induced cardiotoxicity, highlighting Alb as an independent predictor of HF. Collectively, these variables provide a comprehensive basis for assessing individualized HF risk in elderly RRMM patients.

Our nomogram, constructed from four readily available clinical variables, enables individualized HF risk prediction for elderly MM patients receiving carfilzomib-based therapy. The model demonstrated strong discriminative ability and calibration in both internal and external cohorts, supporting its reliability and suitability for routine clinical use. To apply the nomogram, clinicians assign points to each variable based on the nomogram chart, sum these points for a total score, and then convert the score to an absolute risk probability using the accompanying risk table or scale. This practical calculation method, along with representative risk estimates for two patients, is shown in **Table 6** to illustrate clinical application.

A central feature of this model is its threshold-based risk stratification. We selected a 30% probability cutoff to distinguish high- and low-risk patients, based on decision curve analysis and clinical relevance. At this threshold, the model classifies approximately 32.8% of the cohort as high risk, closely matching the actual incidence of HF, and balancing sensitivity with specificity. This stratification helps clinicians focus intensive cardiac monitoring and preventive interventions on patients most likely to benefit. For high-risk patients, early cardiology consultation, baseline and regular cardiac imaging, NT-proBNP monitoring, and aggressive management of modifiable risk factors such as blood pressure and fluid balance are recommended. If additional comorbidities or early signs of cardiac injury are present, clinicians may consider adjusting carfilzomib dosing or exploring alternative regimens. High-risk patients may also benefit from intensified cardiac monitoring, and novel therapies such as sodium-glucose co-transporter 2 (SGLT-2) inhibitors to mitigate carfilzomib-related cardiotoxicity (51). By contrast, patients classified as low risk can generally proceed with standard management and routine monitoring, minimizing unnecessary interventions and treatment burden.

It is important to note that this nomogram was developed specifically for elderly RRMM patients treated with carfilzomib. Carfilzomib is associated with a significantly higher incidence of HF compared with the orally administered ixazomib or intravenous reversible PI such as bortezomib, due to its irreversible inhibition of the proteasome and greater impact on myocardial stress and remodeling (52). As a result, the model’s predictions are most reliable for carfilzomib-treated patients, and its broader applicability to other PI-regimens or MM populations requires further external validation in multicenter studies and real-world datasets.

Carfilzomib-induced HF is characterized by a modest reduction in LVEF and elevated NT-proBNP levels, but both have limitations for early detection. LVEF typically does not reflect early myocardial dysfunction, as detectable changes only appear when a substantial degree of myocardial injury has already occurred and exceeded the sensitivity threshold of LVEF (53). Although NT-proBNP is essential for diagnosing acute HF and monitoring congestion, modest elevations may not be sufficient to predict early cardiac dysfunction due to confounding factors such as steroid use, hydration status, and renal function. The nomogram developed in this study provides an additional tool for risk assessment before clinical symptoms or significant biomarker changes become apparent, which may assist in early monitoring and clinical decision-making in high-risk elderly RRMM patients.

This study has several limitations: (1) The findings are based on data from a single medical center with a limited number of participants; (2) Some factors such as individual health backgrounds (e.g., frailty status), ambulatory blood pressure monitoring, or serological markers like NT-proBNP were not fully captured in our analysis. Although the nomogram performed well in both internal and external validation, further studies using multicenter prospective data or large administrative datasets will be important to confirm its broader utility. The integration of novel biomarkers and advanced cardiac imaging techniques may also improve predictive accuracy in future models.

## 5 Conclusion

In conclusion, this study provides a practical tool for cardiovascular risk assessment in elderly RRMM patients receiving carfilzomib-based therapy, highlighting the importance of Alb levels, hypertension, renal function, and CAD. By using complementary machine learning, we identified consistent predictors and developed a nomogram model with good performance in terms of discrimination, calibration, and clinical utility. This approach can assist clinicians in recognizing higher-risk patients and tailoring monitoring and management strategies throughout the course of carfilzomib treatment.

## Data Availability

The raw data supporting the conclusions of this article are not publicly available due to privacy/ethical restrictions. Processed or anonymized data may be made available from the corresponding author upon reasonable request and with appropriate institutional approvals.
